# Variability of HIV-1 Genomes among Children and Adolescents from São Paulo, Brazil

**DOI:** 10.1371/journal.pone.0062552

**Published:** 2013-05-07

**Authors:** Sabri Saeed Sanabani, Rodrigo Pessôa, Ana Carolina Soares de Oliveira, Vanessa Pouza Martinez, Maria Teresa Maidana Giret, Regina Célia de Menezes Succi, Karina Carvalho, Claudia Satiko Tomiyama, Douglas F. Nixon, Ester Cerdeira Sabino, Esper Georges Kallas

**Affiliations:** 1 Clinical and Research Laboratory (LIM 03), School of Medicine, University of São Paulo, São Paulo, Brazil; 2 Virology Laboratory (LIM 52-HCFMUSP), Institute of Tropical Medicine, University of São Paulo, São Paulo, Brazil; 3 Two Story Lab, Miller School of Medicine, University of Florida, Miami, Florida, United States of America; 4 Department of Pediatrics, Paulista School of Medicine, Federal University of São Paulo, São Paulo, Brazil; 5 Division of Clinical Immunology and Allergy, School of Medicine, University of São Paulo, São Paulo, Brazil; 6 Division of Experimental Medicine, Department of Medicine, University of California San Francisco, San Francisco, California, United States of America; Institut Pasteur of Shanghai, Chinese Academy of Sciences, China

## Abstract

**Background:**

Genetic variability is a major feature of the human immunodeficiency virus type 1 (HIV-1) and considered the key factor to frustrating efforts to halt the virus epidemic. In this study, we aimed to investigate the genetic variability of HIV-1 strains among children and adolescents born from 1992 to 2009 in the state of Sao Paulo, Brazil.

**Methodology:**

Plasma and peripheral blood mononuclear cells (PBMC) were collected from 51 HIV-1-positive children and adolescents on ART followed between September 1992 and July 2009. After extraction, the genetic materials were used in a polymerase chain reaction (PCR) to amplify the viral near full length genomes (NFLGs) from 5 overlapped fragments. NFLGs and partial amplicons were directly sequenced and data were phylogenetically inferred.

**Results:**

Of the 51 samples studied, the NFLGs and partial fragments of HIV-1 from 42 PBMCs and 25 plasma were successfully subtyped. Results based on proviral DNA revealed that 22 (52.4%) patients were infected with subtype B, 16 (38.1%) were infected with BF1 mosaic variants and 4 (9.5%) were infected with sub-subtype F1. All the BF1 recombinants were unique and distinct from any previously identified unique or circulating recombinant forms in South America. Evidence of dual infections was detected in 3 patients coinfected with the same or distinct HIV-1 subtypes. Ten of the 31 (32.2%) and 12 of the 21 (57.1%) subjects with recovered proviral and plasma, respectively, protease sequences were infected with major mutants resistant to protease inhibitors. The V3 sequences of 14 patients with available sequences from PBMC/or plasma were predicted to be R5-tropic virus except for two patients who harbored an X4 strain.

**Conclusions:**

The high proportion of HIV-1 BF1 recombinant, coinfection rate and vertical transmission in Brazil merits urgent attention and effective measures to reduce the transmission of HIV among spouses and sex partners.

## Introduction

Since the beginning of the HIV/AIDS pandemic, until the end of 2010, over 3 million children under 15 years of age have been infected with HIV-1 and 390,000 new viral infections each year (most recent data from UNAIDS/WHO; http://www.who.int/hiv/pub/progress_report2011/hiv_full_report_2011) have been reported. Up until 2009, the Centers for Disease Control and Prevention (CDC) estimates that perinatal transmission of the infection by the mother accounts for 91% of all AIDS cases among children under the age of 13 (CDC- Basic Statistics. Available at: http://www.cdc.gov/hiv/topics/surveillance/basic.htm. Accessed November 21, 2011). While mother to child transmission (MTCT) has been drastically reduced (1–2%) in rich countries, pregnant women living with HIV in poorer countries still have limited access to the same quality of counseling and antiretroviral therapy (ART) [1]. Therefore, these women are at higher risk of transmitting the virus to their offspring during pregnancy, labor or after childbirth via breastfeeding. Risk factors associated with MTCT include lack of receipt of prenatal ART, advanced maternal clinical status, detectable maternal viral load at delivery, low maternal CD4 T cell counts, immunogenetic host factors, and a high viral heterogeneity in the mother [2], [3], [4], [5], [6]. Without treatment, the chance of transmitting HIV from a mother to a baby is somewhere between 12% and 25% in resource rich settings, and between 20% and 45% in resource poor settings [7]. Although MTCT is being addressed by interventions of highly active ART (HAART), which usually comprises three drugs, the ultimate solution to HIV/AIDS will be a globally effective vaccine to curb HIV from spreading further. However, the development of such vaccines requires an in-depth knowledge of the virus strains being transmitted in the target population.

One of the most prominent features of HIV-1 is the remarkable accumulation of genetic diversity in its population during the course of infection. This diversity reflects the high mutation rate of reverse transcriptase (3×10^−5^ substitutions per site per generation) [8], rapid viral turnover (10^−8^ to 10^−9^ virions per day) [9], large number of infected cells (10^7^ to 10^8^ cells) [10], and recombination [11]. Consequently, the HIV-1 population is composed of a swarm of highly genetically related variants, i.e. a *quasispecies*, capable of rapidly adapting to various selective pressures. This diversity has been shown to have an impact not only on viral phenotypes at the level of transmission patterns, pathogenicity and immunology but also in responses to ART and vaccines [12], [13], [14]. Nine distinct genetic subtypes, (A–D, F–H, J and K) are joined in the pandemic today by more than 50 major circulating recombinant forms (CRFs) [http://www.hiv.lanl.gov/content/sequence/HIV/CRFs/CRFs.html] and numerous unique recombinant forms (URFs) have been isolated from individual patients [15], [16].

Brazil, the most populous country in the Latin America, is home to about one third of the people living with HIV (608,230) in Central and South America (UNAIDS. 2010–2011 Report on the Brazilian response to HIV/AIDS; http://www.unaids.org/en/dataanalysis/knowyourresponse/countryprogressreports/2012countries/UNGASS_2012_ingles_rev_08jun.pdf). According to 2008 estimates from UNAIDS (most recent data), 13,728 cases of HIV-infected Brazilian children were notified and 84.5% of them were infected by vertical transmission. Despite the availability of free HIV diagnostic tests and ART, perinatal transmission of HIV-1 infection by the mother remains a national public health challenge in some areas, where difficulties in providing the requisite antenatal HIV screening exist. Like in other European countries and in North America, HIV-1 subtype B is a major genetic clade circulating in the country but the overall prevalence of non-B strains, particularly URF BF1, C and URF BC, is increasing [17], [18], [19], [20]. Data from recent studies of the viral near full length genomes (NFLG) have provided evidence of Brazilian CRF strains designated as CRF28_BF, CRF29_BF, CRF39_BF, CRF40_BF, CRF46_BF and CRF31_BC [18], [19], [21], [22] (http://www.hiv.lanl.gov/content/sequence/HIV/CRFs/CRFs.html.). However, most HIV molecular variability studies in Brazil so far have been limited to adult patients and/or are based on sequence information derived from small genetic fragments that may not predict the subtype classification of other regions of the viral genomes.

Unlike HIV-1 infection in adults, multiple HIV-1 strains are not expected to co-circulate in children and the precise timing of the transmission event can be traced to the time of birth, providing a unique opportunity to explore the viral evolution [23].

The present study was undertaken to describe the genetic variability and identify resistance-associated mutations in HIV-1 isolates recovered from children, adolescents, and young adults with majority perinatally acquired HIV infection.

## Materials and Methods

### Study Population

A cross sectional study was conducted among 51 HIV-1 infected children (ages 0–14 years) and adolescents (aged 15–20 years) on ART followed between September 1992 and July 2009 at the Division of Pediatric Infectious Disease Clinic (CEADIPe), at the Federal University of São Paulo (UNIFESP), Brazil. All, but three of the participants had been born to HIV-1 seropositive mothers. From their medical records, almost all of the seropositive mothers had multiple sexual partners or had an exclusive sexual relationship with a partner whom they knew either to have had other sexual partners or, less commonly, to have a history of injection drug use. Three participants were born to women seronegative for HIV-1 and thus had an unknown transmission mode of their infection. All study participants gave written informed consent. Parents or legal guardians provided written informed consent on behalf of the children. The study plan and consent procedures were approved by the ethics committee of the federal University of São Paulo.

### Amplification & Sequencing

Proviral DNA and RNA were extracted from peripheral blood mononuclear cells (PBMC) and plasma with commercial kits (QIAamp DNA Blood mini Kit and QIAamp Viral RNA Kit, QIAGEN, Germany) according to the manufacturer’s instructions. To make complementary DNAs, the extracted RNA samples were subjected to reverse transcription PCR using SuperScript III (Invitrogen, Carlsbad, CA). Both cDNAs and proviral DNAs were used as the PCR template, as this allowed amplification of the NFLGs from five overlapping fragments as previously described [24], [25], [26]. Amplification reactions were done in duplicate to eliminate PCR artifacts, ensuring that sequenced full-length genomes were not assembled from heterogeneous DNA targets. The expected sizes of the amplified products were verified using ethidium-bromide staining after agarose gel electrophoresis. Each PCR included a known HIV-1 subtype B positive control and an interspersed no DNA template negative controls. Strict laboratory precautions were taken to avoid cross contamination.

Both DNA complementary strands were sequenced directly from purified PCR products in an overlapping fragment of 400 nucleotides by using a variety of sequence specific primers, fluorescent-dye terminators, and *Taq* polymerase on an automated sequencer (ABI 3100, Applied Biosystems Inc., Foster City, CA). The data from the sequenced fragments were edited, assembled into contiguous sequences on a minimum overlap of 20 bp with a 85%–90% minimal mismatch and a consensus of both strands was formed by the Sequencher program (Gene Code Corp., Ann Arbor, MI). Such assembly criteria would prevent any fragment from overlapping if it is not derived from the same variant. All the sequences were checked for contamination by BLAST search against HIV-1 sequence database and among themselves [27].

### Phylogenetic Analysis

Full genome sequences were aligned with reference sequences representing subtypes A–D, F–H, J and K obtained from the Los Alamos database (http://hiv-web.lanl.gov) using the CLUSTAL X program [28] with the “slow-accurate” default alignment parameters and IUB DNA weight matrix. Aligned sequences were manually edited and trimmed to the minimal shared length in the BioEdit Sequence Alignment Editor Program. The gap-stripped aligned sequences were screened for the presence of recombination patterns by the bootscan methods implemented in the SIMPLOT program v3.2 beta 29,30 and the jumping profile Hidden Markov Model [31]. For the bootscan method, nucleotide distances were calculated in a sliding window of 300 bp moving in steps of 30 bp by the F84 model of evolution, transition\transversion ratio of 2.0. Recombinant regions of the alignment as determined by the crossover points from bootscanning were analyzed separately by phylogenetic analysis. Maximum likelihood phylogenies were constructed using the GTR+I+G substitution model and a BIONJ starting tree. Heuristic tree searches under the ML optimality criterion were performed using the NNI branch-swapping algorithm. The approximate likelihood ratio test (aLRT) based on a Shimodaira-Hasegawa-like procedure was used as a statistical test to calculate branch support. The maximum composite likelihood in MEGA version 4 [32] was used to calculate the genetic distances between and within isolates.

### Genotyping Analysis

All amino acid positions associated with ART resistance, in the protease (Pro) and reverse transcription (RT) regions, according to IAS-USA 2011 and Stanford HIV drug resistance database were evaluated on both plasma and blood samples.

### Measurement of HIV RNA and Cell Count

The viral load was measured using the Roche Amplicor HIV-1 Monitor test (Roche, Branchburg, NJ; lower limit of detection 50 copies per ml). CD4+ and CD8+ T cell counts were performed using a lymphocyte staining panel containing CD3, CD4, CD8, and CD45 conjugated monoclonal antibodies (BD Biosciences, San Diego, California, USA).

### Genotypic Tropism Analysis

For the predictions of HIV tropism, the *env* region identified in the NFLGs and partial-length *env* sequences that would encompass the V3 region were analyzed using a tropism prediction algorithm implemented as the web-based service geno2pheno [coreceptor] http://www.geno2pheno.org. To minimize the number of false predictions of CXC chemokine receptor 4 (CXCR4 or X4) tropic sequences as C–C chemokine receptor 5 (CCR5 or R5) tropic, a conservative false-positive rate (FPR) of 20% was used as a cutoff. Therefore, X4 or X4 dual/mixed-tropic viruses (X4/DM) were reported positive if their sequences had a prediction result FPR of ≤20% or the 11/25 rule predicted a X4 virus, otherwise, they were considered R5-tropic viruses.

All nucleotide sequences obtained during our study were reported to GenBank (Accession numbers pending).

## Results

### Samples

In total, 49 paired samples of whole blood and plasma and 2 unpaired samples of each type from an additional 4 different patients were subjected to NFLG amplification and sequencing. Of these 51 subjects, 21 (41.2%) were males and 30 (58.8%) were females. The participants were predominantly white (69.6%), were followed from birth and reached a median age of 11.5 years (range between 4 and 20 years). The median HIV-1 viral load and CD4 cell count, as judged by levels at the time of inclusion, were 6.34×10^2^ copies/ml (range,<49−7.5×10^5^) and 640 cells/mm3 (range, 18–1821 cells/mm3), respectively. Four (8%) patients were naïve and an additional 2 (4%) patients were not taking therapy at the time of enrollment, although they were drug-experienced. The therapeutic status was not known for one subject. Among the 43 subjects, 69.7% had received ≥3 different ART regimens during their follow-up. The median duration of ART at the time of genotyping was 27.5 months. The main characteristics and ART regimens of the study population are given in Table 1.

**Table 1:** Patient characteristics for the study population.

Age, median (range) years	11.5 (4–20)
**Gender (%)**	
Male	21 (41.2)
Female	30 (58.8)
**Race (%)**	
White	32 (69.6)
Black	6 (13)
colored	8 (17.4)
Undetermined	6 (13)
**HIV RNA level, median (range)**	6.34×10^2^ (<49−7.5×10^5^)
Current HIV RNA <50 (%)	19 (36.5)
Current HIV RNA >50 (%)	33 (63.5)
CD4 cell count (cells/mm3), Median (range)	640 (18–1821)
CD8 cell count (cells/mm3), Median (range)	1052 (212–2377)
**Antiretroviral therapy exposure**	
Naïve (%)	4 (7.7)
Previous exposure (%)	2 (3.8)
Unkown status	1 (1.9)
Receiving antiretroviral therapy (%)	45 (86.6)
**Treatment regimen at the time of enrollment**	
Combined NRTIs+PI (%)	28 (62.2)
Combined NRTIs (%)	4 (8.9)
Combined NRTIs+NNRTI (%)	9 (20)
NRTI+NNRTI+ PI (%)	3 (6.6)
NRTI+PI (%)	1 (2.3)

### NFLG and Partial Amplification of HIV-1 from both PBMC and Plasma Specimens

Sequences were obtained for all five overlapped fragments that cover the NFLGs of 4 PBMC DNA and one plasma RNA virus. Partial sequences were obtained from at least one fragment derived from 38 blood and 24 plasma samples as shown in Table 2. Of the 26 plasma samples for which partial and NFLGs failed, 21 (80.7%) had a viral load under 500 copies/ml and the remaining 5 (19.3%) RNA viruses had multiple peaks present in the sequencing chromatogram probably indicating different *quasispecies* in the same sample or HIV-1 dual infections. On the other hand, partial amplification of 1126 bp of fragment B1(Nucleotide position from start of HXB2 genome 2196–3322) and 494 bp stretch of fragment D (Nucleotide position from start of HXB2 genome; 8997–9491) were subtype B positive for isolates 010BR_IMT_010 and 010BR_IMT_051, respectively, and both patients had viral loads below 50 copies/ml (Table 2). These results may suggest an underestimation of the measured viremia or high efficacy of our nested PCR approach in some patients. On the other hand, our results among paired samples demonstrated that 20 patients had detectable HIV proviral DNA and undetectable viral RNA, 21 were dually positive for viral RNA and DNA, and 4 patients were dually negative. It is unclear why we were unable to amplify more plasma RNA viruses, particularly for patients 010_BR_IMT_05, 010_BR_IMT_12, 010_BR_IMT_54, and 010_BR_IMT_58 (median viral load 1.5×10^4^, range 1.6×10^3^–2.9×10^4^) using our fragment-based amplification strategy, although RNA degradation may account for this finding.

**Table 2:** The near full-length genomic (NFLG) and partial fragments subtyping of HIV-from plasma and blood samples.

Sequence ID	Sample	Age/yrs	Sequence fragment					Subtype	VL*
			A _(546–2598)_	B1_ (2157–3791)_	B2_ (3236–5220)_	C _(4890–7808)_	D _(7719–9537)_		
010BR_001	PBMC	20	–	–	–	–	–		
	Plasma		–	–	–	–	–		49
010BR_002	PBMC	14	–	–	–	–	–		
010BR_IMT_002_pl	Plasma		–	+	–	–	–	BF1	7433
010BR_003	PBMC	19	–	–	–	–	–		
010BR_IMT_003_pl	Plasma								
010BR_004	PBMC	17	–	–	–	–	–		
010BR_IMT_004_pl	Plasma								
010BR_005	PBMC	7	+	+	–	+	+	BF1	
010BR_IMT_005_pl	Plasma		–	–	–	–	–		5473
010BR_006	PBMC	11	–	+	–	+	+	BF1	
010BR_IMT_006_pl	Plasma		+	–	–	+	–	BF1	1543
010BR_007	PBMC	11	–	–	–	+	–	B	
010BR_IMT_007_pl	Plasma		–	–	–	–	–		49
010BR_008	PBMC	11	–	–	–	–	–		
010BR_IMT_008_pl	Plasma		–	–	–	–	–		49
010BR_009	PBMC	8	+	–	–	+	+	B	
010BR_IMT_009_pl	Plasma		+	–	–	–	–	B	1879
010BR_010	PBMC	13	–	+	–	–	–	B	
010BR_IMT_010_pl	Plasma		–	+	–	–	–	B	49
010BR_011	PBMC	10	–	+	–	–	–	F1	
010BR_IMT_011_pl	Plasma		+	–	–	–	–	BF1	1452
010BR_012	PBMC	10	–	–	–	–	+	B	
010BR_IMT_012_pl	Plasma		–	–	–	–	–		29361
010BR_013	PBMC	12	–	–	–	+	–	BF1	
010BR_IMT_013_pl	Plasma		+	–	–	–	–	B	560
010BR_014	PBMC	11	–	–	–	–	–		
010BR_IMT_014_pl	Plasma		+	–	–	–	–	F1	1858
010BR_015	PBMC	11	+	–	–	–	–	B	
010BR_IMT_015_pl	Plasma		+	–	–	+	–	B	3859
010BR_016	PBMC	7	+	+	–	+	–	BF1	
010BR_IMT_016_pl	Plasma		+	+	–	–	–	B	33650
010BR_017	PBMC	18	–	–	–	+	+	B	
010BR_IMT_017_pl	Plasma		–	–	–	+	–	B	39153
010BR_018	PBMC	15	+	–	–	–	–	B	
010BR_IMT_018_pl	Plasma		–	–	–	–	–		49
010BR_019	PBMC	11	+	–	–	–	–	B	
010BR_IMT_019_pl	Plasma		+	–	–	–	–	B	1209
010BR_020	PBMC	14	+	+	+	+	+	B	
010BR_IMT_020_pl	Plasma		–	–	–	+	–	B	572
010BR_021	PBMC	12	+	–	–	–	+	B	
010BR_IMT_021_pl	Plasma		–	–	–	–	–		87
010BR_022	PBMC	10	+	–	–	+	–	BF1	
010BR_IMT_022_pl	Plasma		–	–	–	–	–		49
010BR_023	PBMC	10	–	–	–	–	–		
010BR_IMT_023_pl	Plasma		–	–	–	–	–		49
010BR_025	PBMC	13	–	–	–	+	+	BF1	
010BR_IMT_025_pl	Plasma		–	–	–	–	–		49
010BR_026	PBMC	11	+	+	+	+	+	BF1	
010BR_IMT_026_pl	Plasma		+	–	–	+	–	B	37134
010BR_027	PBMC	7	+	–	–	–	+	B	
010BR_IMT_027_pl	Plasma		–	–	–	+	–	B	10038
010BR_029	PBMC	14	+	+	–	–	+	BF1	
010BR_IMT_029_pl	Plasma		–	–	–	–	–		49
010BR_030	PBMC	15	–	–	–	–	+	BF1	
010BR_IMT_030_pl	Plasma		–	–	–	–	–		49
010BR_031	PBMC	15	+	–	–	–	–	F1	
010BR_IMT_031_pl	Plasma		–	–	–	–	–		49
010BR_032	PBMC	13	–	+	+	–	–	B	
010BR_IMT_032_pl	Plasma		–	+	+	+	–	B	7824
010BR_033	PBMC	10	+	–	–	–	+	B	
010BR_IMT_033_pl	Plasma		–	–	–	–	–		49
010BR_034	PBMC	10	+	+	–	–	–	B	
010BR_IMT_034_pl	Plasma		+	–	–	–	–	B	13391
010BR_035	PBMC	14	–	–	–	–	+	BF1	
010BR_IMT_035_pl	Plasma		+	+	+	+	+	BF1	35172
010BR_036	PBMC	18	–	–	–	+	+	B	
010BR_IMT_036_pl	Plasma		+	–	–	–	+	B	17292
010BR_037	PBMC	4	+	–	–	–	–	B	
010BR_IMT_037_pl	Plasma		+	–	–	–	–	B	697
010BR_038	PBMC	11	–	–	–	–	+	B	
010BR_IMT_038_pl	Plasma		–	–	–	–	–		365
010BR_039	PBMC	12	+	–	–	–	+	B	
010BR_IMT_039_pl	Plasma		+	–	–	–	–	B	83187
010BR_040	PBMC	16	–	+	–	–	–	BF1	
010BR_IMT_040_pl	Plasma		–	–	–	–	–		49
010BR_041	PBMC	9	+	+	+	+	+	F1	
010BR_IMT_041_pl	Plasma		+	+	+	+	–	BF1	5411
010BR_042	PBMC	10	+	+	+	–	+	BF1	
010BR_IMT_042_pl	Plasma		+	+	–	–	–	B	13213
010BR_043	PBMC	9	–	–	–	+	–	B	
010BR_IMT_043_pl	Plasma		–	–	–	–	–		49
010BR_044	PBMC	19	–	+	+	+	–	B	
010BR_IMT_044_pl	Plasma		–	–	–	–	–		750000
010BR_045	PBMC	14	+	–	–	+	+	BF1	
010BR_IMT_045_pl	Plasma		–	–	–	–	–		49
010BR_046	PBMC	6	+	–	–	–	+	BF1	
010BR_IMT_046_pl	Plasma		–	–	–	–	–		139
010BR_047	PBMC	11	+	+	+	+	+	F1	
010BR_IMT_047_pl	Plasma		+	+	+	+	–	F1	15556
010BR_048	PBMC	13	–	–	–	–	–		
010BR_IMT_048_pl	Plasma		+	–	–	–	+	F1	10365
010BR_049	PBMC	14	–	+	+	–	+	BF1	
010BR_IMT_049_pl	Plasma		–	–	–	–	–		49
010BR_051	PBMC	ND							
010BR_IMT_051_pl	Plasma		–	–	–	–	+	B	49
010BR_054	PBMC	13	–	+	–	–	+	B	
010BR_IMT_054_pl	Plasma		–	–	–	–	–		24453
010BR_056	PBMC	11							
010BR_IMT_056_pl	Plasma		–	–	–	–	–		49
010BR_057	PBMC	13	+	+	–	–	+	B	
010BR_IMT_057_pl	Plasma		–	–	–	–	–		49
010BR_058	PBMC	14	+	+	–	–	–	BF1	
010BR_IMT_058_pl	Plasma		–	–	–	–	–		1671
010BR_060	PBMC	12	–	–	–	–	–		
010BR_IMT_060_pl	Plasma		–	–	–	–	–		126

* VL – Viral Load (copies/mL).

### HIV Variants and Sequences

**Figure 1:**
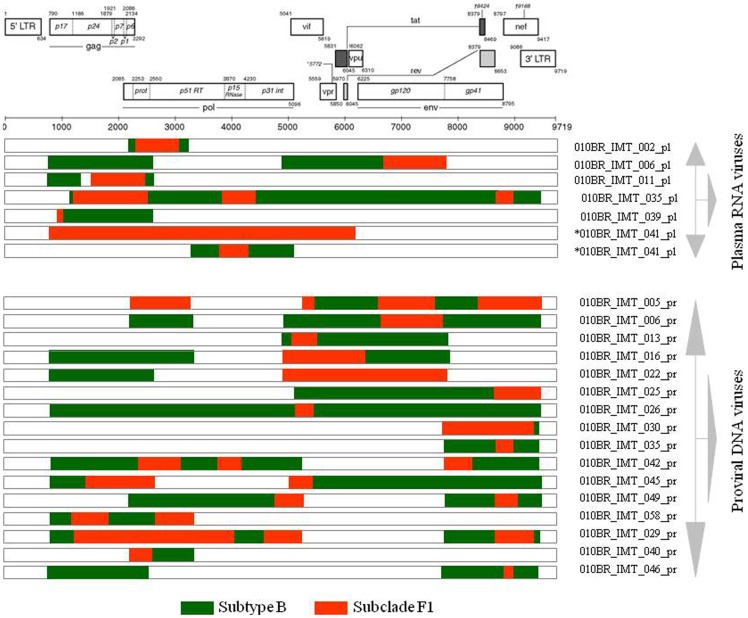
Schematic representation of the NFLG, partial structure and breakpoint profiles of the BF1 sequences identified in this study both from HIV RNA and proviral DNA. Samples that were identified in this study to host distinct viruses are indicated with the star symbol. The region of subclade F1 and subtypes B are indicated at the bottom.

**Figure 2:**
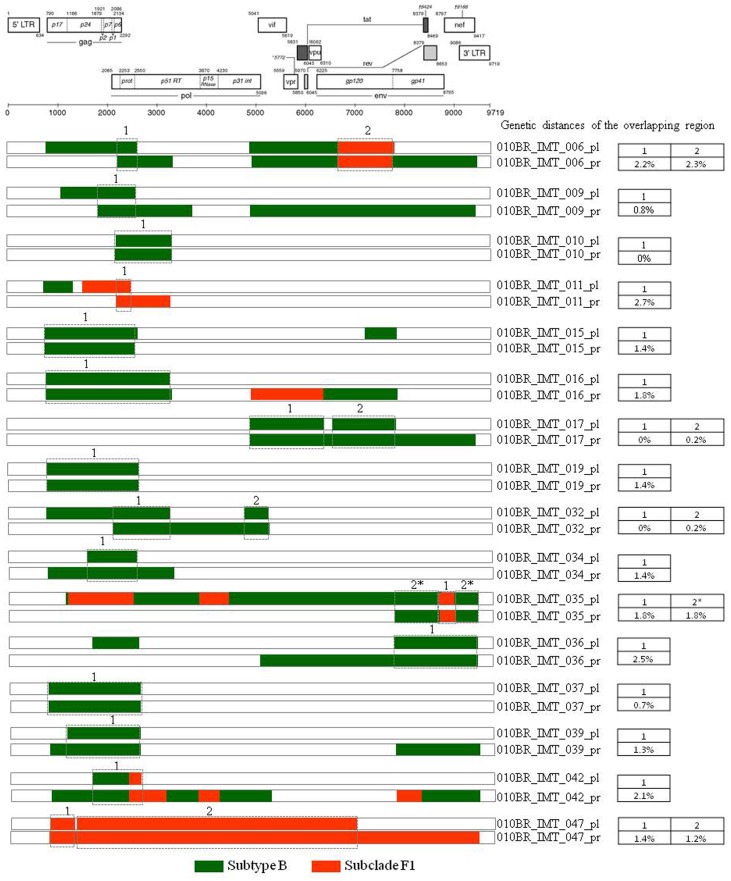
Genetic distances of overlapping regions between isolates recovered from patients with paired plasma and PBMC samples. Concatenated sequences are indicated with the star symbol. The region of subclade F1 and subtypes B are indicated at the bottom.

**Figure 3:**
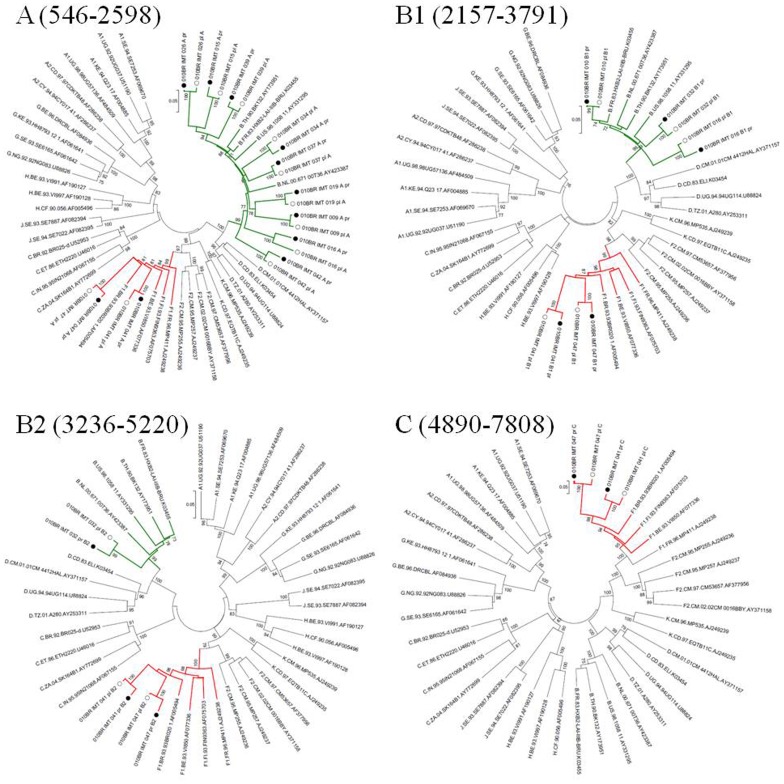
Maximum-likelihood phylogenetic trees form each non-recombinant fragment were constructed using all available sequences from proviral DNA (indicated by black circles) and plasma isolate (indicated by an empty circles) along with HIV-1 reference sequences from the Los Alamos HIV-1 database representing 11 genetic subtypes. The numbering for the HIV-1 fragment A, B1, B2 and C sequences corresponds to the HXB2 reference sequence. For purposes of clarity, the tree was midpoint rooted. The approximate likelihood ratio test (aLRT) values of ≥70% are indicated at nodes. The scale bar represents 0.05 nucleotide substitutions per site.

**Figure 4:**
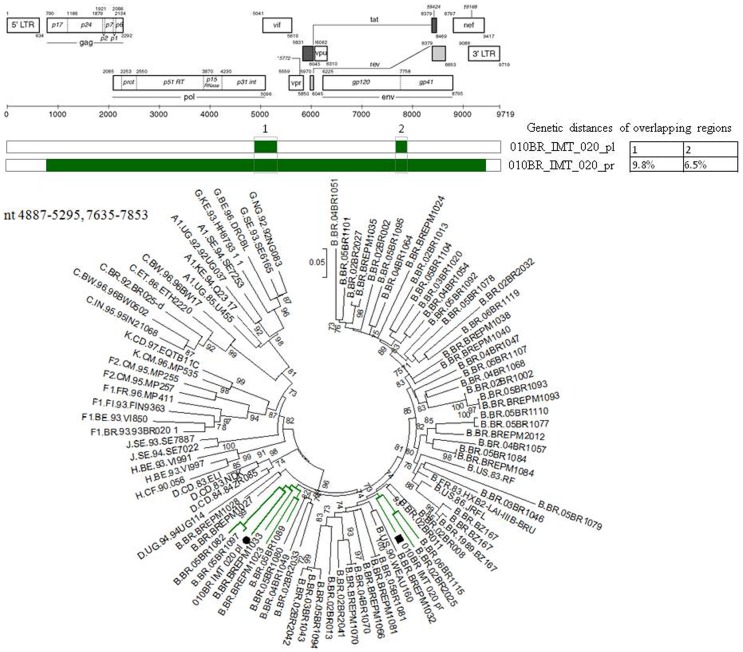
Genetic distances and phylogenetic tree constructed using a maximum-likelihood method from concatenated regions of HIV RNA (indicated by black circles) and proviral DNA (indicated by black squares) marked with Arabic numbers 1 and 2 from patient 010BR_IMT_020 along with HIV-1 reference sequences from the Los Alamos HIV-1 database representing 11 genetic subtypes. For purposes of clarity, the tree was midpoint rooted. The approximate likelihood ratio test (aLRT) values of ≥70% are indicated at nodes. The scale bar represents 0.05 nucleotide substitutions per site.

**Figure 5:**
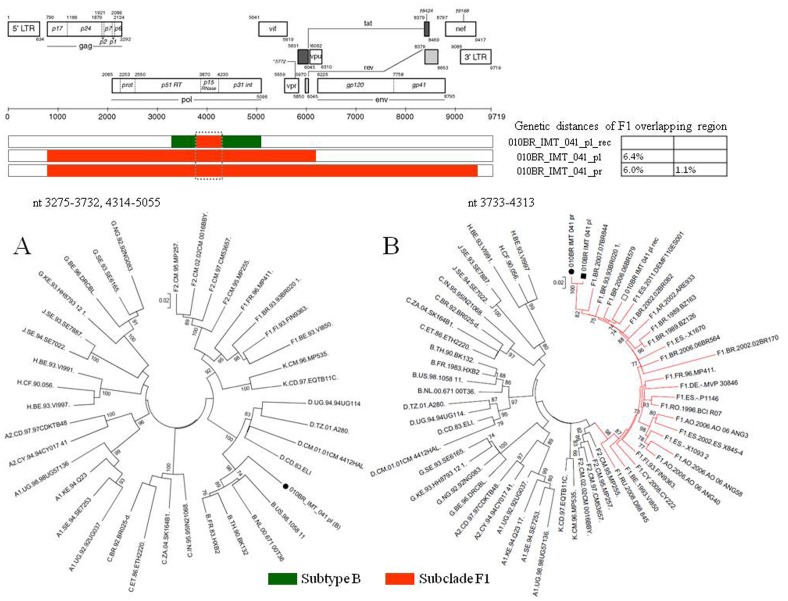
Genetic distances and phylogenetic tree constructed using a maximum-likelihood method of HIV isolates recovered from patient 01BR_IMT_041. A: Phylogenetic tree from concatenated regions assigned as subtype B from the BF1 recombinant isolate. B: Phylogenetic tree showing the clustering pattern of F1 sequences (marked by dotted box). F1 region from genuine F1 sequence recovered from plasma and PBMCs are marked by a black circle and square, respectively while the F1 region from the BF1 recombinant sequence is marked by an empty square. The approximate likelihood ratio test (aLRT) values of ≥70% are indicated at nodes. The scale bar represents 0.05 nucleotide substitutions per site.

**Figure 6:**
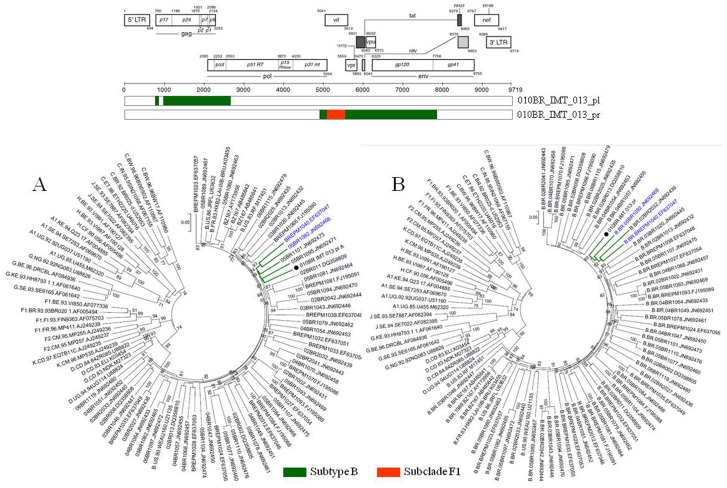
Phylogenetic clustering profile of the non-overlapped fragments assigned as subtype B from both plasma and provirus isolate 01BR_IMT_013 were compared to a number of additional Brazilian subtype B sequences and other HIV-1 reference sequences from the Los Alamos HIV-1 database representing 11 genetic subtypes. For purposes of clarity, the tree was midpoint rooted. The approximate likelihood ratio test (aLRT) values of ≥70% are indicated at nodes. The scale bar represents 0.05 nucleotide substitutions per site.

Based on phylogenetic analysis, the NFLGs and partial proviral nucleotide sequences (*n* = 42) of the clinical HIV-1 isolates indicated that 22 (52.4%) patients were infected with HIV-1 subtype B, 16 (38.1%) were infected with a mosaic consisting of subtype BF1 and 4 (9.5%) were infected with sub-subtype F1 (Table 2). Of the total 25 plasma samples for which viral subtype was determined, 17 (68%) were classified as subtype B, 3 (12%) were sub-subtype F1, and 5 (20%) were BF1 recombinant viruses (Table 2). All chimeric viruses were unique according to their recombination profile, i.e., not assigned to any subtype or CRF (Figure 1). The relationships of the viral sequences from patients’ PBMCs to the sequences obtained from the corresponding RNA virus within the same regions were examined for each patient to assess the viral diversity in both compartments. The results revealed that all but one patient, 010BR_IMT_020, had plasma RNA and proviral DNA variation only ranging between 0–2.7% (Figure 2). These relations were further confirmed by phylogenetic analysis, which showed close branching as demonstrated in Figure 3. These findings may indicate that the primary infected PBMCs of these patients were likely the source of plasma circulating viral sequences however; more sophisticated genetic tests able to detect viral population structure are needed to confirm this conclusion. The observed differences in the percent nucleotide variations between proviruses and plasma free viruses in this group may reflect evolution that occurs during the initial phase of acute infection, before the therapeutic control of HIV-1 replication is established. Surprisingly, the intra-individual plasma and proviral sequence variation for patient 010BR_IMT_020 in the overlapped regions depicted in Figure 4 were 9.8% and 6.5%, respectively, indicating that the plasma viruses were derived from a population significantly distinct from those of the cellular sources in this 13 years old asymptomatic patient. This result is consistent with dual distinct variants of the same subtype being involved in establishing infection. Dual infection with subclade F1 and BF1 recombinant was observed in patient 010BR_IMT_041 plasma sample (Figure 5). This patient was a nine year old child who diagnosed in February 2005 and until the sampling period had been asymptomatic. The patient had been receiving ART since September 2005. This observation of dual infection occurred accidentally during assembling of the generated data, in which some sequences failed to assemble to other overlapping stretches of fragment B1. As a result, we sought to compare this stretch to HIV sequences available from public databases. Upon analysis with the basic local alignment search tool (BLAST) available from GenBank, the stretch (010BR_IMT_041_PL- REC; 548 bp) from plasma revealed high percentages of nucleotide sequence identity to the BF1 isolate 99JY-TRA0133 (Genbank accession: JN235964), whereas the other larger fragment (010BR_IMT_041_pl; 5720 bp) revealed high homology to subclade F1 isolate 02BR082 (Genbank accession: FJ771006) at the nucleotide levels. To ensure that the generation of the two consensus sequences from patient 010BR_IMT_041 plasma sample was not the result of sample contamination, repeat sequence analysis using the purified B1 amplicon was performed and revealed identical findings. These results possibly indicate that some internal sequencing primers of fragment B1 preferentially annealed to the BF1 string during sequencing reaction. Regions that were the same F1 subclade in the two *pols* were then compared to determine whether the 010BR_IMT_041_PL viruses were the actual parents of the recombinant fragment or if an infection in this patient was acquired with two genetically distinct viruses (Figure 5B). While both partial *pol* genes were sub-subtype F1 fragments, these were from different subclade F1 isolates, since the sequences from the two plasma demonstrated high nucleotide divergence (up to 6.8%). Moreover, as shown in Figure 5B, both F1 non-recombinant sequences recovered from plasma and PBMC clustered separately (aLRT 100%) and the branch lengths separating them from the F1 fragment involved in the recombination event were typical for other sequences of unrelated F1 variants. The analysis was then extended to include isolates with non-overlapping fragments, namely 010BR_IMT_013 and 010BR_IMT_027, to determine whether the PBMC viruses were truly parental strains to those recovered from the plasma. For this purpose, the phylogenetic clustering profile of the non-overlapped fragments from both compartments were compared to a number of additional Brazilin subtype B and other HIV-1 reference sequences to increase our confidence in the analyses and provide a broader perspective. These results revealed the magnitude of aLRT value supporting the identical clustering of the plasma isolate 010BR_IMT_013_pl (Figure 6A) and the proviral 010BR_IMT_013_pr strains (Figure 6B) with the Brazilian subtype B BREPM 1040 and 05BR 1092 subtype B sequences (branch marked with green color). Based on these results, it is possible to assume that the primary infected PBMCs in this patient were likely the source of the plasma circulating viral sequences. However, this interpretation does not hold true when the analysis was applied to the plasma and proviral non-overlapping fragments of patient 010BR_IMT_027. The clustering profile to subtype B references and genetic distances as shown in Figure S1 were significantly different between both fragments in this patient indicating dual infection with distinct subtype B variants.

### Genotypic Drug Resistance Test Results

The results of the genotyping analysis from both plasma RNA and whole blood DNA are presented in Table 3 and 4. Resistance analyses were performed from proviral DNA in 32 patients (19 PR/RT and 13 PR) and from plasma-associated RNA in 21 patients (8 PR/RT and 13 PR). Regardless of paired or unpaired samples, 10 of the 31 (32.2%) and 12 of the 21 (57.1%) subjects with recovered proviral and plasma PR sequences, respectively, were on ART at the time of specimen collection and were infected with major mutants resistant to protease inhibitors (PIs). Regarding the primary resistant mutations for PIs among the naive patients with available PR sequences from PBMCs (*n* = 4) and plasma (*n* = 3), various mutations were detected in only one patient (010BR_IMT_034; Table 3 and 4). The RT region of the provirus of the same patient displayed some major transmitted mutations both for the NRTIs and NNRTIs. Detailed frequency of single Pro and RT mutations detected in patients on ART both in plasma RNA and whole blood DNA is also illustrated in Table 3 and 4.

**Table 3:** Drug-resistance mutations detected in plasma.

Sample ID	Resistance mutations			HIV-1 subtype	Tropism	CV*
	PI	NRTI	NNRTI			
010BR_IMT_002	M36I	**M184V**		BF1		7433
010BR_IMT_006	L10V, K20R, M36I, **I54V**, L63P, A71V,**V82A**, **L90M**			BF1	R5	1543
010BR_IMT_009	K20T, M36I, I62V, L63P, I64V,**V82I**, **L90M**			B		1879
010BR_IMT_011	L10V, K20R, L24I, M36I, **M46L**, **I54V**,L63P, V82A			BF1		1452
010BR_IMT_013	I64V, V77I, **V82I**			B		560
010BR_IMT_014	M36I, **L89M**			F1		1858
010BR_IMT_015	M36I			B	R5	3859
010BR_IMT_016	L63P			B		33650
010BR_IMT_019	D60E, I62V, L63P, I64V			B		1209
010BR_IMT_026	M36I, L63P			B	R5	37134
010BR_IMT_032	D60E, L63P, H69K, V77I	**M184V**		B	R5	7824
010BR_IMT_034	L10V, **V32I**, L33F, K43T, **M46I**, **I54V**,L63P, A71V, **V82A**, L89V, **L90M**			B		13391
010BR_IMT_035	L10V, K20T, M36I, I62V, L63P, L89I,**L90M**, I93L	**M41L**, A62V, V75I, F116Y, **Q151M**, **K65R**	**G190A**	BF1	X4	35172
010BR_IMT_036	L10I, L33F, **M36I**, **I54V**, Q58E, D60E,I62V, I64V, **L76V**, **V82A**, L89M			B		17292
010BR_IMT_037	I62V, I64V			B		697
010BR_IMT_039	L63P, A71V, V77I, I93L			B		83187
010BR_IMT_041	L10V, K20R, M36I, **M46I**, **I54V**, I62V,L76V, **V82A**, L89M	**M41L**, **L74V**,**M184V**, **T215Y**	**K103N**	F1		5411
010BR_IMT_042	L10I, K20R, M36I, Q58E, D60E, L63P,A71V, **V82A**, **L90M**, I93L			B		13213
010BR_IMT_047	L10V, M36I, **L89M**		**K103N**	F1	R5	15556
010BR_IMT_048	M36I, I64V			F1		103665

* copies/Ml.

Regions not sequenced are indicated by empty boxes.

High resistance mutation are indicated by bold lettering.

**Table 4:** Drug-resistance mutations detected in PBMC.

Sample ID	Resistance mutations			HIV-1 subtype	Tropism
	PI	NRTI	NNRTI		
010BR_IMT_005	L10V, L20R, M36I	**M41L**, **T215Y**		BF1	R5
010BR_IMT_006	**I54V**, **V82A**, L10V, L20R, M36I,L63P, A71V, I93M	**M41L**,V75M, **M184V**, **T215Y**	**K103N**, A98G,V108I, H221Y	BF1	R5
010BR_IMT_009	K20T, M36I, I62V, L63P, I64V,**V82I**, **L90M**			B	R5
010BR_IMT_011^1^	L24I, **I54V**, **V82A, L90M**, L10I,L20R, M36I, L63P	**D67G**,**K70R**, **M184V**		F1	
010BR_IMT_015	M36I			B	
010BR_IMT_016	M36I, L63P		**G190A**, **E138A**	BF1	R5
010BR_IMT_018	M36I, I64V, H69K			B	
010BR_IMT_019	D60E, I62V, L63P, I64V			B	
010BR_IMT_020	M36I, **M46I**			B	R5
010BR_IMT_021	I64V			B	
010BR_IMT_022	I64V			BF1	R5
010BR_IMT_026	M36I, L63P	**M41L**,**T215C**		BF1	R5
010BR_IMT_027	A71V, V77I, I93L			B	
010BR_IMT_029	**V82A**, L20R, M36I	**D67N**, K70R, **M184V**		BF1	
010BR_IMT_031	M36I, L63P			F1	
010BR_IMT_032	D60E, L63P, H69K, V77I	**M184V**		B	
010BR_IMT_033	G16E, L33V, I62V, V77I			B	
010BR_IMT_034	**I30V**,**M46I**, **I54V**, **V82A**,L33F, A71V	**D67N**, **K70R**, **L210W**, **T215Y**		B	
010BR_IMT_037	I62V, I64V			B	
010BR_IMT_039	L63P, A71V, V77I, I93L			B	
010BR_IMT_040	**D30N**, **M46I**, **I54V**, **L76V**, **V82A**, **L90M**, L10I, L20R, M36I, I62V	**M41L**, **D67N**, **M184V**	**E138K**	BF1	
010BR_IMT_041	**M46I**,**I54V**, **L76V**, **V82A**, L10V,L20R, M36I	**M41L**, **L74V**, V75M, **M184V**, **T215Y**	**K103N**	F1	X4
010BR_IMT_042	**I54L**, **V82A**, **L90M**, L10I, M36I,Q58E, D60E, L63P, A71V, V77I, I93L	**M41L**, **D67N**,L74I, V75T	**Y181C**	BF1	
010BR_IMT_045	M36I			BF1	R5
010BR_IMT_046	M36I			BF1	
010BR_IMT_047	**M46I**, L10V, M36I			F1	R5
010BR_IMT_049	I64V, V77I, I93L	**L210W**, **T215Y**	**Y181C**	BF1	
010BR_IMT_054	**L90M**, L20I, M36I, L63P, A71T	**M41L,** **M184V**, **T215Y**	**L100I**, **K103N**	B	
010BR_IMT_057	**D30N**, M36I, L63P			B	
010BR_IMT_058	M36I, D60E, I62V, I64V	**M41L**, **M184V**, **T215Y**	**K103N**,P225H,**E138A**	BF1	

1 Displayed insertion at position 69.

Regions not sequenced are indicated by empty boxes.

High resistance mutations are indicated by bold lettering.

### V3 Sequence Analysis and Viral Tropism

An evaluation of the V3 loop amino acids and predictions of viral tropism were performed for patients with available sequences from PBMCs (*n* = 10) and plasma (*n* = 7) of the derived fragment C intact frame sequences. The inferred HIV tropism in paired samples of plasma and PBMCs was successfully determined in 3 samples and all concordant with the R5 virus. The inferred HIV tropism study in the other 3 plasma demonstrated that 2 patients harbored the R5 virus. The V3 sequences of the 7 patients with available sequences from only PBMC were predicted to be R5-tropic virus except for patient 01BR_IMT_035 who harbored an X4 strain.

## Discussion

This study describes the genetic variability and the prevalence of drug resistance mutations and co-receptor usage of HIV-1 variants in a small, well sampled group of children and adolescents. The majority of these patients acquired their infection through vertical transmission during the period 1992–2007. The results presented confirmed that subtype B is still the main HIV-1 variant and concordant with data from other studies on adult and children populations from Brazil [24], [33], [34], [35]. The most remarkable observations in this study are that at least 38.1% of the 42 patients with proviral DNA sequences are infected with HIV-1 BF1 recombinant variants, which is relatively much higher if compared to earlier studies on children and adolescent patients in Brazil [33], [34], [35], [36], [37], [38]. This difference is not surprising, because small fragments from different regions of HIV genomes were characterized in the previous studies while we used larger overlapped fragments to sequence the full-length genome, which undoubtedly provides efficient discrimination of HIV subtypes and the recombinant forms. Thus, the earlier study is likely to have missed some recombinants. Despite the high rate of recombination in our study, it is probable that our results have also underestimated the true rate of infection with BF1 recombinant viruses, particularly among patients with partially sequenced viral fragments. Thus, it is possible that the BF1 infection in this group may be higher than what was observed if we had sequenced the virus NFLG in all samples. Our attempts to amplify the NFLG or additional larger fragments for some samples to determine if recombination had occurred were unsuccessful. Other likely explanations for underestimation of BF1 recombination rate is that some isolates could have been undetected by our PCR strategy because of a mismatch at the primer binding sites, low proviral load, employment of consensus sequences or that the BF1 isolates were maintained in another reservoir other than the CD4-positive compartment that was sampled in the peripheral blood. The results that indicate none of the BF1 recombinant structures identified in this study showed any similarity to the known CRFs or other recombinants strongly suggests that new recombinants are arising continually in São Paulo, Brazil.

Additional observations of this study are the description of the high level of intra-host diversity with evidence of mixed infections with the same or distinct HIV-1 subtypes. The observation that patients may be simultaneously infected with different HIV-1 subtypes has been reported in numerous cases and considered of significant interest. For instance, the first documented dual infection of two distinct HIV-1 subtypes B and E (later designated as CRF01_AE) was reported in Thailand [39]. Janini *et al.* [40] reported the first case of both horizontal and subsequent vertical transmission of 2 distinct HIV-1 subtypes from 1 dually infected person to another. In the present study, dual infections were evident in three patients, lending further support to previous studies [41], [42], [43], as this event is far more common in Brazil where both subtypes co-circulate. The fact that existence of dual infection in some patients contrasts with the hypothesis that an initial viral infection produces some degree of protection against a second infecting HIV subtype. If we assume that super-infection occurs in these cases, then it is conceivable that antiviral immunity evoked by one subtype had insufficient broad protection at the time of primary infection against a second infecting virus. Indeed this assumption has been challenged previously by convincing findings revealing that a second super-infection with a different HIV-1 strain can occur long after an initial infection is established and can hasten the disease process [44], [45]. Alternatively, the subjects 010BR_IMT_041 and 010BR_IMT_027 (confirmed MTCT) may have been vertically and concomitantly infected with different HIV strains at the same time. On the basis of this assumption, our results may suggest that despite the genetic bottleneck occurring upon vertical transmission of HIV-1, the replication capacity of transmitted variants is not necessarily reduced. This interpretation is in line with previous studies that provided evidences of multiple-variant transmission in MTCT, and also agreed with the conclusion that in a majority of cases the infant is infected with a single isolate [46], [47], [48]. By the lack of mode of transmission in patient 010BR_IMT_020, it was therefore not possible to interpret the simultaneous detection of both viruses.

Our results on genotype resistance mutations are consistent with previous studies with similar subjects indicating that prevalence of major mutations conferring ART resistance in viral DNA/RNA of such chronically infected groups is common (20/38, 52.6%) [34]. In patients with available sequences from the Pro and/or RT, the mutations found in PBMCs were generally also found in the plasma, although some of the patients showed few differences between the two compartments, while in one patient (010BR_IMT_011) the 69 insertion in the protease region was found in PBMC, but not in plasma.

Regardless of the sample compartment, the analysis of HIV tropism revealed two patients with X4 viruses and both with CDC class “C3” reflecting advanced disease. The assessment of HIV tropism in our study was limited to sequence- based algorithms rather than using phenotypic methods. Although phenotypic assays still have an edge over genotypic methods, genotypic predictors prove to be highly concordant with phenotype data and can reliably be used to determine viral tropism with better results in PBMC than in plasma samples [49]. In this study, we used geno2pheno, which has shown a similar performance to the Trofile phenotypic assay, the most often used tropism method [50]. Moreover, the method has been shown to achieve higher sensitivity while retaining high level of specificity when compared with the performance of different algorithms [51], [52].

We are aware that the demonstration of the high recombination rate and evidence of double infections and their association with virological response and viral tropism should be based on a larger dataset to establish statistical influence of these factors in determining the outcome. Another limitation of this study is that direct bulk sequencing and genotyping of HIV-1 in plasma and whole blood might underestimate low-level minority species present as *quasispecies* which could be evidenced by more complex methods, such as massive parallel pyrosequencing [53], [54], [55]. Despite these limitations, the results of this analysis indicate that HIV-1 recombination and dual infections are much more frequent than thought previously among children and adolescents in this region. Evidently, more extensive studies with large sample sizes are required to unravel the mechanisms underlying the emergence of these recombinants and their implications for HIV control.

## Supporting Information

Figure S1 **Comparison of phylogenetic clustering profile of the fragments assigned as subtype B from both plasma and provirus isolate 01BR_IMT_027 were compared to a number of additional Brazilian subtype B sequences and other HIV-1 reference sequences from the Los Alamos HIV-1 database representing 11 genetic subtypes.** For purposes of clarity, the tree was midpoint rooted. The approximate likelihood ratio test (aLRT) values of ≥70% are indicated at nodes. The scale bar represents 0.05 nucleotide substitutions per site.
(TIFF)
