# Supplementary figures and images for: Variability of HIV-1 Genomes among Children and Adolescents from São Paulo, Brazil
Source: PLoS One. 2013 May 7;8(5):e62552. doi: 10.1371/journal.pone.0062552 (PMC3646872; doi:10.1371/journal.pone.0062552)

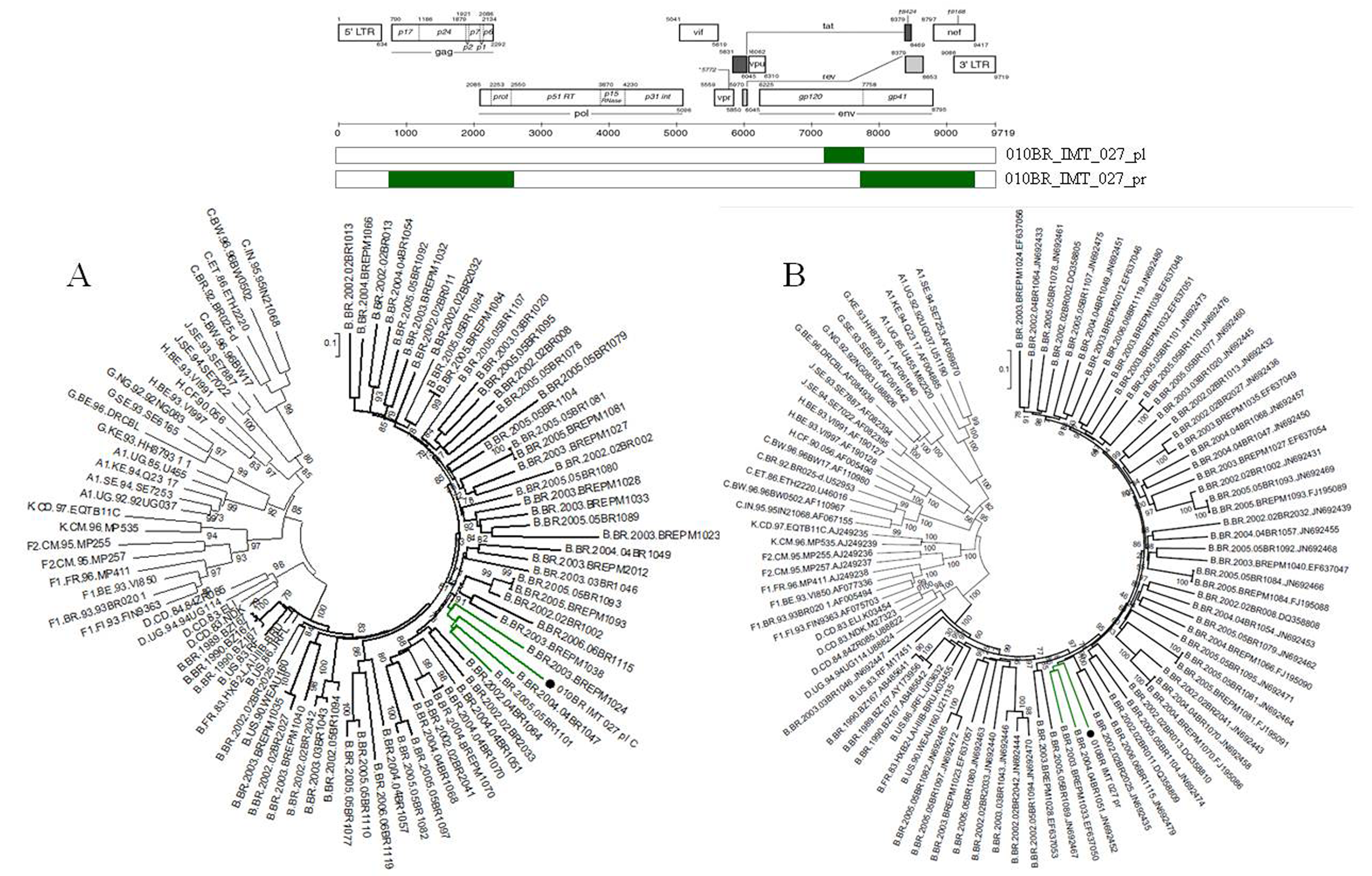

Supplement: Figure S1 — Comparison of phylogenetic clustering profile of the fragments assigned as subtype B from both plasma and provirus isolate 01BR_IMT_027 were compared to a number of additional Brazilian subtype B sequences and other HIV-1 reference sequences from the Los Alamos HIV-1 database representing 11 genetic subtypes. For purposes of clarity, the tree was midpoint rooted. The approximate likelihood ratio test (aLRT) values of ≥70% are indicated at nodes. The scale bar represents 0.05 nucleotide substitutions per site. (TIFF) [file pone.0062552.s001.tiff]
